# Importance of County and City Boards of Health

**Published:** 1912-02

**Authors:** H. W. Terrell


### IMPORTANCE OF COUNTY AND CITY BOARDS OF HEALTH.

By H. W. Terrell.

In presenting this paper I shall spring nothing new, nor demand any special Board of Health laws, but if I can succeed in arousing the professions and laity to the importance and almost crying necessity of well organized and active County and City Boards of Health, I shall feel repaid.

After several attempts to organize a State Board of Health, it was not till 1903 that the Georgia Legislature aroused from its sleep and passed the State Board of Health bill. I don't know whether it was the great interest they felt in protecting humanity, or lack of something else to protect, as long before this act was passed bills had been passed protecting cattle, horses, birds and fish. Having nothing else to protect except man, wild beasts and reptiles, they passed a bill entitled, "An Act to Establish the State Board of Health, etc." and made the and maintain it.

But we should be thankful for all small blessings and large ones in proportion, for subsequent legislatures have generously given: in 1904, $3,000.00; in 1905, $4,364.69; in 1906, $7,500.00; in 1907, $7,500.00; 1908, $16,000.00; in 1909 $16,000.00; 1910, $21,500.00; 1911, $30,500.00 and this to furnish diphtheria antitoxin and for the establishment of a Pasteur Institute.

Think of this wholesale generosity—$109,864.09 for ten years' protection of two million, seven hundred thousand human beings from disease and death. This 10 years' appropriation is about the amount appropriated for the Agricultural Department of the State University for the year 1911. This being done, contented they seemed to say, "Glory to God in the highest, and in Georgia, peace and good health to man."

When a few years ago, the State Board of Health asked for $5,000.00 to investigate the hook-worm disease, they were met with cold shoulders and rebuffs from the medical members of the House, yet when $5,000.00 was asked for by the Agricultural Department for hog cholera, and $5,000.00 for cattle tick, the members of the legislature fell over themselves to see that this protection was given.

We, as medical men, should not confine our studies entirely to the art of medicine, but better prepare ourselves in the science of medicine. The duty of the physician is not simply to administer to the sick, for frequently his duties are ended there by the death angel. But his duty is to prevent as far as possible many of the contagious and infectious diseases.

This can be done chiefly, and I might say only, through well-organized and active County and City Boards of Health.

In this movement the laity naturally look to the medical profession for advice and suggestion.

Unfortunately, there is in the medical profession too little harmony along this line and the laity will naturally follow the advice of the family physician in preference to the more rigid rules and instructions of the local Board of Health, as he is regarded as an authority on medical matters, a veritable encyclopedia.

If we would familiarize ourselves more with the bacteriology of diseases, we could frequently better advise our clientele along lines of sanitation and hygiene and thereby preventing the spread of many of the contagious and infectious diseases. I had hoped to present you a tabulated report of the sanitary condition of this section of Georgia, with special reference to the schools. The replies to questions sent to the Mayors, County School Commissioners, Superintendents of Public Schools and Secretaries of County Medical Societies were so at variance that it was well-nigh impossible to do, so I abandoned the idea.

Ex-President Roosevelt has said: "The health of our citizens is the greatest asset of the nation, our health departments, municipal, State and National, should be regarded as an extremely important branch of the public service."

From the evidence I could get, it seems that it is not so regarded in this section of Georgia, for few cities and fewer counties in Georgia have Boards of Health and those are inactive.

have but little jurisdiction, and are provided for in a miserly way, if at all.

Commercialism, or the fear of hurting business in a financial way, seems to prevent some of our cities and towns from reporting epidemics and taking active steps to prevents its spread. No thought seems to be taken of the health and lives of the unwise and unsuspecting citizen.

Prof. Irving Fisher, of Yale University, estimates the productive value of life at $1,700.00. Estimating the population of Georgia at 2,700,000 and taking the $30,500.00 appropriated for looking after their health, shows that it is spending, in 1912 one and one-tenth cents ($.01 1-10) for your individual protection against disease.

In 1911 Georgia made the magnificent appropriation of $2,550,000.00 to the common schools, which is a little over $4.00 per pupil, according to report of State School Commissioner. While I would not detract one iota from the sentiment favoring public schools and education, nor subtract one cent from the appropriation, I do submit that an education head with a diseased body is worth but little more, if any, than a healthy body with an undeveloped mind.

We are proud of the rapid and great growth of education and I would be the last man in this Society to decry it, but when we consider the great suffering and great number of deaths from preventable diseases, it does seem it is getting more than its share in proportion to public health and sanitation.

Statistics show that in 48 large cities of the United States, in 1907, the ratio of health department expenses to the total government is 1.8 per cent. of the total disbursement.

The total disbursements for Georgia in 1910 was $5,956,-104.95, showing her appropriation for the Health Department is a little more than one-half of one per cent. of the total.

This shows how little interest the large cities of the United States and the State of Georgia are taking in one of their most valuable assets—human lives.

From information obtained from State School Commissioner's report for 1910, I am able to give the following statistics: We have in Georgia, 7,946 rural schools, containing 555,794 pupils. These rural schools, of course, are all supplied with open closets and but few of these use disinfectants, a few do disinfect occasionally, others not at all.

This does not refer to the city schools and colleges. While some of these have waterworks, a large number of them use open closets, and only four of five of these schools, and these in large cities, have medical inspectors or directors.

I am unable to give statistics of the city of high schools in this section, but find only one school having medical inspection and that inspection done by the Sanitary Committee of the City Council.

I am unable to give an accurate number of the various epidemics we all know to have existed, but from information from our State Board of Health, I am advised that only five or six have been reported since its existence.

"Section 5, of the Act establishing the State Board of Health is as follows:

Section 5. Be it further enacted, that it shall be the duty of the local boards of health and of physicians in localities where there are no health authorities, to report to the State Board of Health, promptly upon discovery thereof, the existence of any of such other contagious or infectious diseases as the State Board of Health from time to time may specify, and when any contagious or infectious disease shall become, or threaten to become, epidemic in any county, city, village or hamlet, and the local authorities shall neglect or refuse to enforce sufficient measures for its prevention, the tate Board of Health may appoint a medical or sanitary officer with such assistants as he may require, and it shall be the duty of such officer to enforce the orders or regulations of the State Board of Health."

In the face of the various epidemics, is it not time, Mr. President, that the physicians were bestirring themselves along this line, thereby upholding themselves and the State Board of Health? We would gain more respect for the State Board of Health, the profession and ourselves.

We must remember that there are in Georgia about 2,70000c people practically all at the mercy of all the contagious and infectious diseases that stalk over the country, and it behooves us, the guardian of our people's health, to give out the danger signals and to use our every combined effort to stay the spread of these fatal diseases that so often result from indifference and neglect on the part both of the laity and our profession.

Last, but not least, let us in the name of a neglected humanity, appeal to the lawmaking powers of our great State, to give us more assistance in this great organized work to allay the suffering of her people, and to stay the ravages of the Grim Reaper.

---

Dr. J. M. Poer, West Point, Ga., in complimenting the Doctor on this paper for the time and trouble he had taken in getting statistics and facts, said he felt that the Doctor had taken the right view of the matter. He also felt constrained to compliment the ladies of his home town, West Point, for the earnest efforts they were making along the lines of hygiene in the schools, also through the town. Told of their being instrumental in establishing the individual drinking cup in the school, of the sanitary method they had insisted on being used in the sweeping of the building, etc., and now they wanted a sanitary drinking fount in the school, and as the ladies usually got what they insisted on, he supposed the children would soon have this. He also spoke of the improvements in sanitation throughout the town since they had been so active in their work.

